# Comprehensive prognostic effects of systemic inflammation and Insulin resistance in women with breast cancer with different BMI: a prospective multicenter cohort

**DOI:** 10.1038/s41598-023-31450-w

**Published:** 2023-03-15

**Authors:** Guo-Tian Ruan, Hai-Lun Xie, Chun-Lei Hu, Chen-An Liu, He-Yang Zhang, Qi Zhang, Zi-Wen Wang, Xi Zhang, Yi-Zhong Ge, Shi-Qi Lin, Meng Tang, Meng-Meng Song, Xiao-Wei Zhang, Xiao-Yue Liu, Kang-Ping Zhang, Ming Yang, Kai-Ying Yu, Kun-Hua Wang, Wen Hu, Li Deng, Ming-Hua Cong, Han-Ping Shi

**Affiliations:** 1 Department of Gastrointestinal Surgery/Department of Clinical Nutrition, Beijing Shijitan Hospital, Capital Medical University, Beijing, 100038 China; 2 National Clinical Research Center for Geriatric Diseases, Xuanwu Hospital, Capital Medical University, Beijing, 100053 China; 3 Key Laboratory of Cancer FSMP for State Market Regulation, 10 Tie Yi Road, Beijing, 100038 China; 4 Yunnan University, Kunming, 650091 China; 5 General Surgery Clinical Medical Center of Yunnan Province, Kunming, 650032 China; 6 Clinical Nutrition Department, Sichuan University West China Hospital, Chengdu, 610041 Sichuan China; 7 Comprehensive Oncology Department, National Cancer Center/Cancer Hospital, Chinese Academy of Medical Sciences and Peking Union Medical College, Beijing, 100038 China

**Keywords:** Cancer, Breast cancer, Cancer metabolism, Cancer prevention, Cancer therapy, Cytokines, Inflammation

## Abstract

To investigate the prognostic value of systemic inflammation and insulin resistance in women with breast cancer with different body mass index (BMI). This multicenter, prospective study included 514 women with breast cancer. Multivariate survival analysis showed that patients with high C-reactive protein (CRP), high CRP to albumin ratio (CAR), high lymphocyte to CRP ratio (LCR), high low-density lipoprotein cholesterol to high-density lipoprotein cholesterol ratio (LHR), and high triglyceride to high-density lipoprotein cholesterol ratio (TG/HDL-c) were significantly associated with worse prognosis. The mortality rate of patients with both high CAR and high LHR or both low LCR and high LHR were 3.91-fold or 3.89-fold higher than patients with both low CAR and low LHR or both high LCR and low LHR, respectively. Furthermore, the combination of LCR and LHR significantly predicted survival in patients within the high BMI group. The CRP, CAR, LCR, LHR, and TG/HDL-c were associated with poor survival in women with breast cancer. The combination of CAR and LHR or LCR and LHR could better predict the prognostic outcomes of women with breast cancer, while the combination of LCR and LHR could better predict the prognosis of those patients with overweight or obese patients.

## Introduction

The 2022 cancer statistics for the United States show that from 2014 to 2018, female breast cancer incidence continued to increase (by 0.5% annually), and the number of new female breast cancer cases was 287,850 (31%), ranking as the most prevalent new cancer in women. Female breast cancer had the second highest mortality rate with 43,250 (15%) deaths^1^. In China, the incidence of female breast cancer still the highest among women^2^. Inflammation and insulin resistance (IR) play important roles in a variety of chronic diseases, including cancer^3^. Cancer is generally considered an inflammatory disease, and systemic inflammation is often a hallmark of cancer and a major driver of metabolic alterations in cancer patients^4,5^. The production of acute-phase proteins, such as C-reactive protein (CRP), is considered an accurate measure of systemic inflammation and pro-inflammatory cytokine activity^6^. Glucose intolerance is the earliest identified metabolic abnormality in cancer patients^7^, resulting in a type II diabetic state with IR^8^. Glicksman et al. showed that about 37% of cancer patients have a diabetic glucose tolerance curve^9^. The characteristics of IR in cancer patients are distinct from those in type II diabetic patients, in which normal fasting blood glucose is associated with high, normal, or low insulin levels^10^, manifested by increased hepatic glucose production and gluconeogenesis, possibly due to intracellular gluconeogenesis^11^. The redistribution of glucose to supply energy needs can lead to hypoglycemia, which in turn, leads to an increase in compensatory hormonal signaling or glucagon.

In recent years, obesity has become the most common metabolic disease worldwide, and its incidence has rapidly increased^12^. Unfortunately, obesity is fast becoming an epidemic in developed and many developing countries^13^. Overweight or obesity is associated with an increased risk of recurrence or death in patients with breast cancer^14,15^. Some obesity-related cancers, such as those of the breast and internal organs, occur in or near fat depots. This suggests that altered fat biology, typically found in the context of elevated BMI, locally contributes to the development of several cancers^16^. Obesity-induced inflammation or inflammatory disturbances are a major feature of adipose tissue dysfunction^17^. In fact, adipose tissue is not only a storehouse of excess energy in the form of triacylglycerols (TAGs), but is also an active endocrine organ secreting different peptides called adipocytokines^18^. The production and expression of inflammatory adipocytokines, such as interleukin (IL)-6, tumor necrosis factor α (TNF-α), and monocyte chemoattractant protein 1, are increased in obese and insulin-resistant subjects^19^. Compared with lean people, adipose tissue in obese subjects was inflamed by inflammatory macrophages^20^. Macrophages are important and key contributors to adipocyte inflammation^21^. Inflammatory macrophages typically accumulate within adipose tissue, and this accumulation leads to localized inflammation. This local inflammation leads to multiple metabolic disturbances, including atherosclerosis and systemic inflammation^22^. In addition, CRP, another inflammatory marker, is elevated in the serum of individuals with higher BMI^23^. IR is a common pathological condition in obese patients with impaired insulin action in adipose tissue. During IR, insulin is significantly increased in the circulation to avoid hyperglycemia^24^. Therefore, insulin is included in the study as a hormone, and insulin levels are often increased in the setting of obesity^24^. This hyperinsulinemia is associated with BMI^25^.

Some reports showed that CRP alone^26–28^ or in combination with other inflammatory markers, such as the CRP to albumin ratio (CAR)^29,30^, were associated with poor prognosis in breast cancer. Recently, Lymphocyte to C-reactive Protein Ratio (LCR) has been reported to be related to cancer prognosis^31–33^, but there is no relevant report on the relationship between LCR and breast cancer prognosis. Studies showed that elevated insulin levels and hyperinsulinemia are associated with poor prognosis in breast cancer patients^34,35^. Some simple and feasible IR surrogate indicators reported earlier have attracted attention relative to the homeostasis model assessment of IR (HOMA-IR)^36^. These IR indicators included fasting triglyceride glucose (TyG) index^37,38^, low-density lipoprotein cholesterol to high-density lipoprotein cholesterol ratio (LDL-c/HDL-c, LHR)^39^, triglyceride to high-density lipoprotein cholesterol ratio (TG/HDL-c)^38^, and total cholesterol to high-density lipoprotein cholesterol ratio (TC/ HDL-c)^38^. Thus, in this study, we aimed to select the optimal IR index in breast cancer, and select the best combination of inflammation index and IR index in breast cancer patients with different body mass index (BMI). Finally, we selected the best combination of inflammation and IR indicators for combined survival analysis and selected the best combination of indicators to predict the survival of patients with breast cancer with different BMI. This study aimed to analyze the prognostic value of systemic inflammation and IR markers in women with breast cancer, as well as their distribution and ability to predict survival in different BMI subgroups.

## Results

### Baseline characteristics

**Figure 1:**
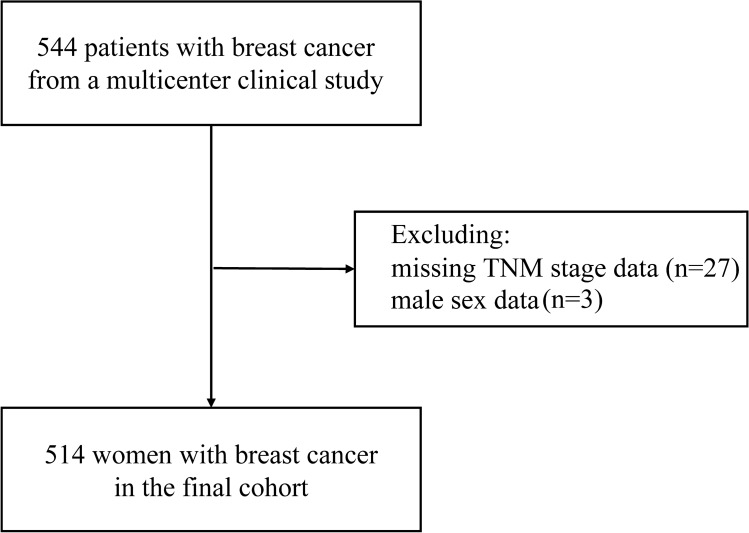
Flowchart of patient selection for this study.

After excluding 3 male breast cancer cases and 27 missing TNM stage data, a total of 514 women with breast cancer were included in our study. The detailed flow chart is showed in Fig. 1. Their mean age was 53.72 ± 10.87 years, and the population’s mean BMI was 24.36 kg/m^2^. Comparing the baseline differences between patients in different BMI groups (low BMI group, BMI < 24 kg/m^2^ vs. high BMI group, BMI ≥ 24 kg/m^2^), the age (54.65 vs. 52.75, *P* = 0.047), BMI (27.23 vs. 21.34, *P* < 0.001), CRP (3.02 vs. 2.78,* P* = 0.020), CAR (0.07 vs. 0.06,* P* = 0.029), TyG (4.55 vs. 4.51,* P* = 0.008), LHR (2.40 vs. 2.09, *P* < 0.001), TG/HDL-c (1.72 vs. 1.34, *P* < 0.001), and TC/HDL-c (4.15 vs. 3.61, *P* < 0.001) were all higher in the patients in high BMI group than those in low BMI group. However, the LCR (6408.0 vs. 5254.9,* P* = 0.029) was higher in the patients in the low BMI group than those in high BMI group. Table 1 shows the baseline characteristics of the women with breast cancer. The median follow-up time for patients was 43.1 (40.7–49.6) months, and the 5-year overall mortality rate was 70 (18%), resulting in 41.4 mortality events per 1000 patient-year.

**Table 1:** Baseline characteristics.

Variables	Overall	BMI < 24 (kg/m^2^)	BMI ≥ 24(kg/m^2^)	*P* value
	(n = 514)	(n = 251)	(n = 263)	
Age (mean (SD))	53.72 (10.87)	52.75 (11.40)	54.65 (10.28)	0.047
BMI (mean (SD))	24.36 (3.80)	21.34 (2.02)	27.23 (2.70)	< 0.001
Tumor stage (%)				0.312
I–II	283 (55.1)	132 (52.6)	151 (57.4)	
III–IV	231 (44.9)	119 (47.4)	112 (42.6)	
Surgery (%)	420 (81.7)	195 (77.7)	225 (85.6)	0.028
Radiotherapy (%)	27 (5.3)	14 (5.6)	13 (4.9)	0.901
Chemotherapy (%)	328 (63.8)	149 (59.4)	179 (68.1)	0.050
Immunotherapy (%)	43 (8.4)	20 (8.0)	23 (8.7)	0.874
KPS (mean (SD))	90.60 (10.29)	89.28 (11.57)	91.86 (8.73)	0.004
Tumor metastasis (%)	42 (8.2)	19 (7.6)	23 (8.7)	0.745
Family history of cancer (%)	100 (19.5)	40 (15.9)	60 (22.8)	0.063
Diabetes (%)	39 (7.6)	19 (7.6)	20 (7.6)	1.000
Hypertension (%)	78 (15.2)	26 (10.4)	52 (19.8)	0.004
CHD (%)	25 (4.9)	8 (3.2)	17 (6.5)	0.128
Lymphocyte, *109/L (mean (SD))	1.62 (1.14)	1.64 (1.48)	1.60 (0.66)	0.643
Albumin (mean (SD))	40.60 (4.80)	40.60 (5.37)	40.59 (4.20)	0.975
CRP (median (IQR))	3.01 (3.61)	2.78 (2.81)	3.02 (3.64)	0.020
LCR (median (IQR))	5806.5 (12,301.7)	6408.0 (13,677.6)	5254.9 (10,204.5)	0.031
CAR (median (IQR))	0.07 (0.09)	0.06 (0.07)	0.07(0.09)	0.029
Glucose (mean (SD))	5.85 (1.95)	5.77 (2.14)	5.93 (1.74)	0.343
TC (mean (SD))	4.88 (1.55)	4.76 (1.57)	5.00 (1.52)	0.081
TG (mean (SD))	1.79 (1.03)	1.61 (1.03)	1.96 (1.00)	< 0.001
HDL-c (mean (SD))	1.32 (0.33)	1.39 (0.36)	1.25 (0.29)	< 0.001
LDL-c (mean (SD))	2.83 (0.79)	2.76 (0.83)	2.89 (0.75)	0.051
TyG (mean (SD))	4.53 (0.19)	4.51 (0.20)	4.55 (0.18)	0.008
LHR (mean (SD))	2.25 (0.73)	2.09 (0.71)	2.40 (0.71)	< 0.001
TG/HDL-c (mean (SD))	1.54 (1.18)	1.34 (1.23)	1.72 (1.10)	< 0.001
TC/HDL-c (mean (SD))	3.89 (1.44)	3.61 (1.33)	4.15 (1.49)	< 0.001
TSF (mean (SD))	22.09 (7.91)	18.97 (7.20)	25.06 (7.42)	< 0.001

*SD* standard deviation, *IQR* interquartile range, *BMI* body mass index, *KPS* karnofsky performance status, *CHD* coronary heart disease, *CRP* C-reactive protein, *CAR* C-reactive protein to albumin ratio, *LCR* lymphocyte to C-reactive protein ratio, *TC* total cholesterol, *TG* triglyceride, *HDL-c* high-density lipoprotein cholesterol, *LDL-c* low-density lipoprotein cholesterol, *TyG* triglyceride-glucose index, *LHR* LDL-c/HDL-c ratio.

### Differences in the distribution of inflammation and IR markers in different BMI subgroups

**Figure 2:**
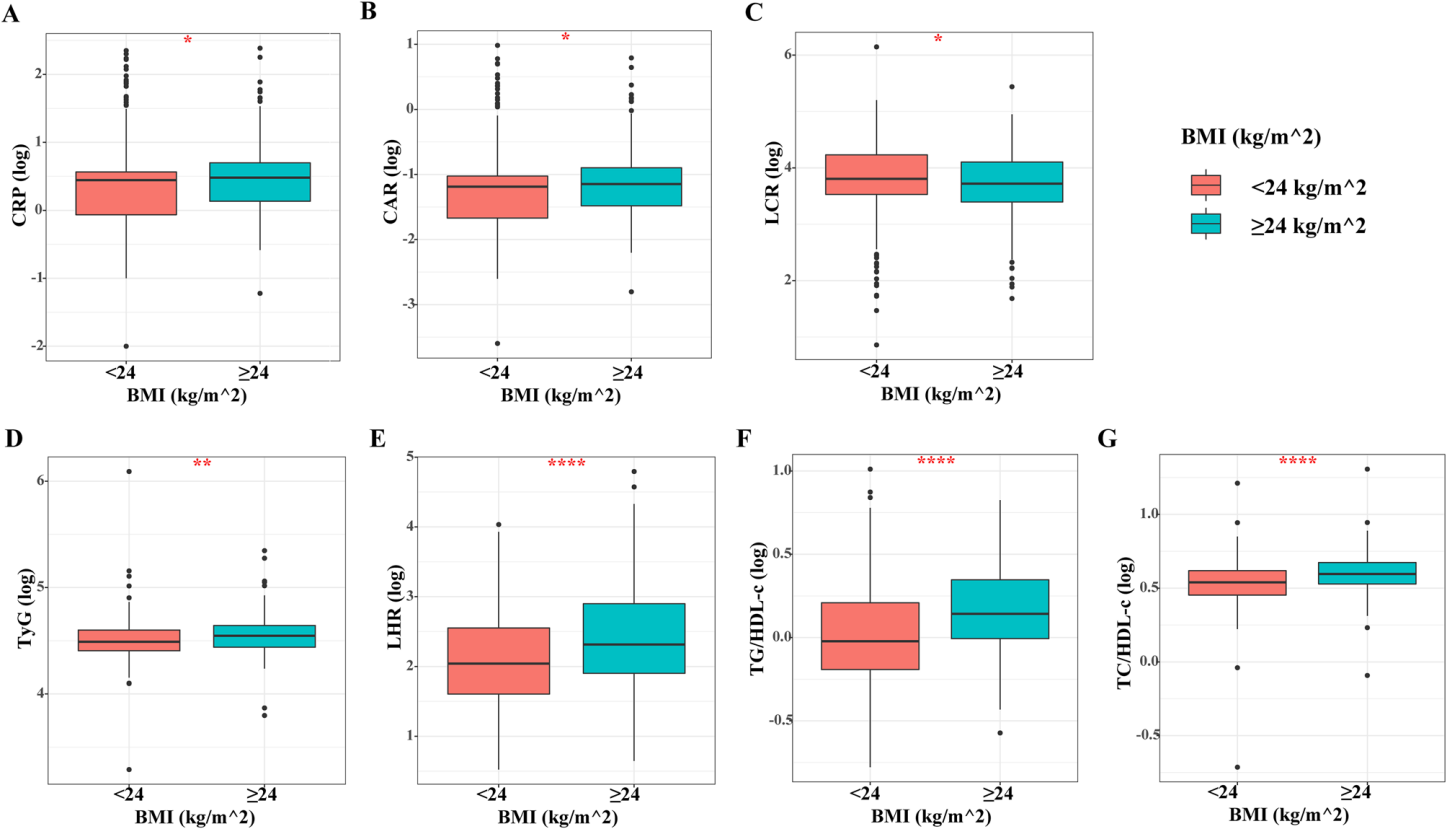
The distribution of systemic inflammatory indicators and IR makers stratified by BMI in women with breast cancer. (**A**) CRP; (**B**) CAR; (**C**) LCR; (**D**) TyG; (**E**) LHR; (**F**) TG/HDL-c; (**G**) TC/HDL-c. *Notes IR* insulin resistance, *BMI* body mass index, *CRP* C-reactive protein, *LCR* lymphocyte to C-reactive protein ratio, *CAR* C-reactive protein to albumin ratio, *TyG* triglyceride-glucose index, *LHR* LDL-c/HDL-c ratio, *HDL-c* high-density lipoprotein cholesterol, *LDL-c* low-density lipoprotein cholesterol.

The distribution curves for systemic inflammation-related indicators in different BMI subgroups showed that the CRP, CAR, and LCR values in the high BMI group were significantly higher than those in the low BMI group (All *P* < 0.05) (Fig. 2A–C). Similarly, we analyzed the differences in the distribution of different IR indicators in different BMI subgroups and found that TyG, LHR, TG/HDL-c, and TC/HDL-c were all highly distributed in the high BMI group compared with the low BMI subgroup patients (All *P* < 0.05) (Fig. 2D–G).

### Prognostic AUC curves and survival analysis correlated with systemic inflammatory markers and IR markers

**Figure 3:**
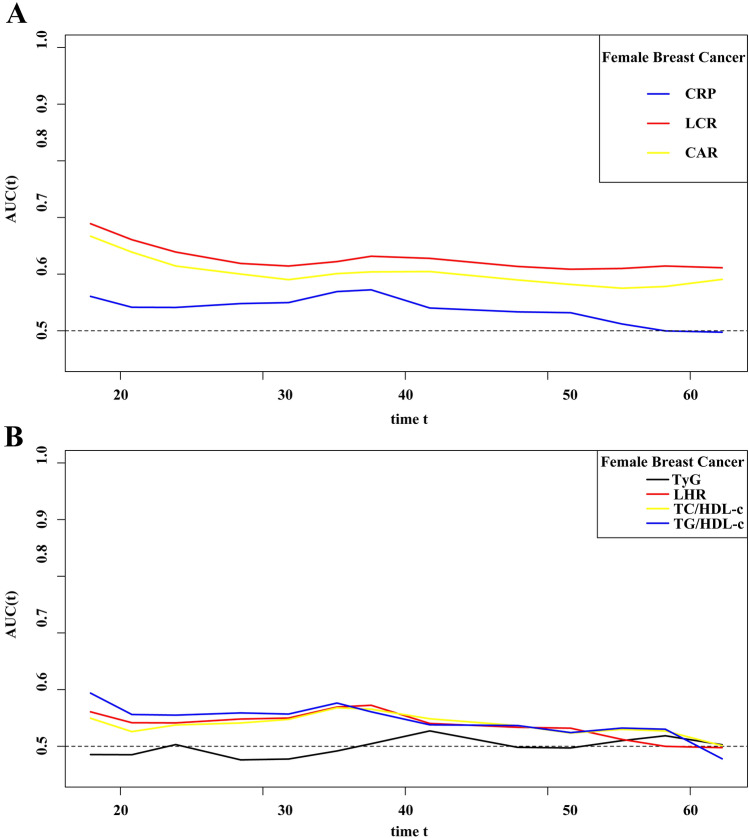
The prognostic AUC curves of systemic inflammatory indicators and IR makers in female breast cancer. (**A**) Systemic inflammatory indicators of CRP, CAR, and LCR; (**B**) IR makers of TyG, LHR, TG/HDL-c, and TC/HDL-c. *Notes AUC* area under the curve, *IR* insulin resistance, *CRP* C-reactive protein, *LCR* lymphocyte to C-reactive protein ratio, *CAR* C-reactive protein to albumin ratio, *TyG* triglyceride-glucose index, *LHR* LDL-c/HDL-c ratio, *HDL-c* high-density lipoprotein cholesterol, *LDL-c* low-density lipoprotein cholesterol.

To select the optimal inflammatory index and IR index in female breast cancer, we drew the prognostic area under the curve (AUC) curves of inflammatory index and IR index, respectively. The results showed that the predictive ability of LCR and CAR was better than that of CRP among different inflammatory indicators, while among different IR indicators, TyG showed the worst predictive ability of prognosis, compared with LHR, TG/HDL-c, and TC/HDL-c (Fig. 3).

**Figure 4:**
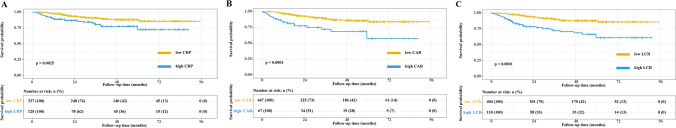
The Kaplan–Meier survival curves of systemic inflammatory indicators in women with breast cancer. (**A**) CRP; (**B**) CAR; (**C**) LCR. *Notes CRP* C-reactive protein, *LCR* lymphocyte to C-reactive protein ratio, *CAR* C-reactive protein to albumin ratio.

**Figure 5:**
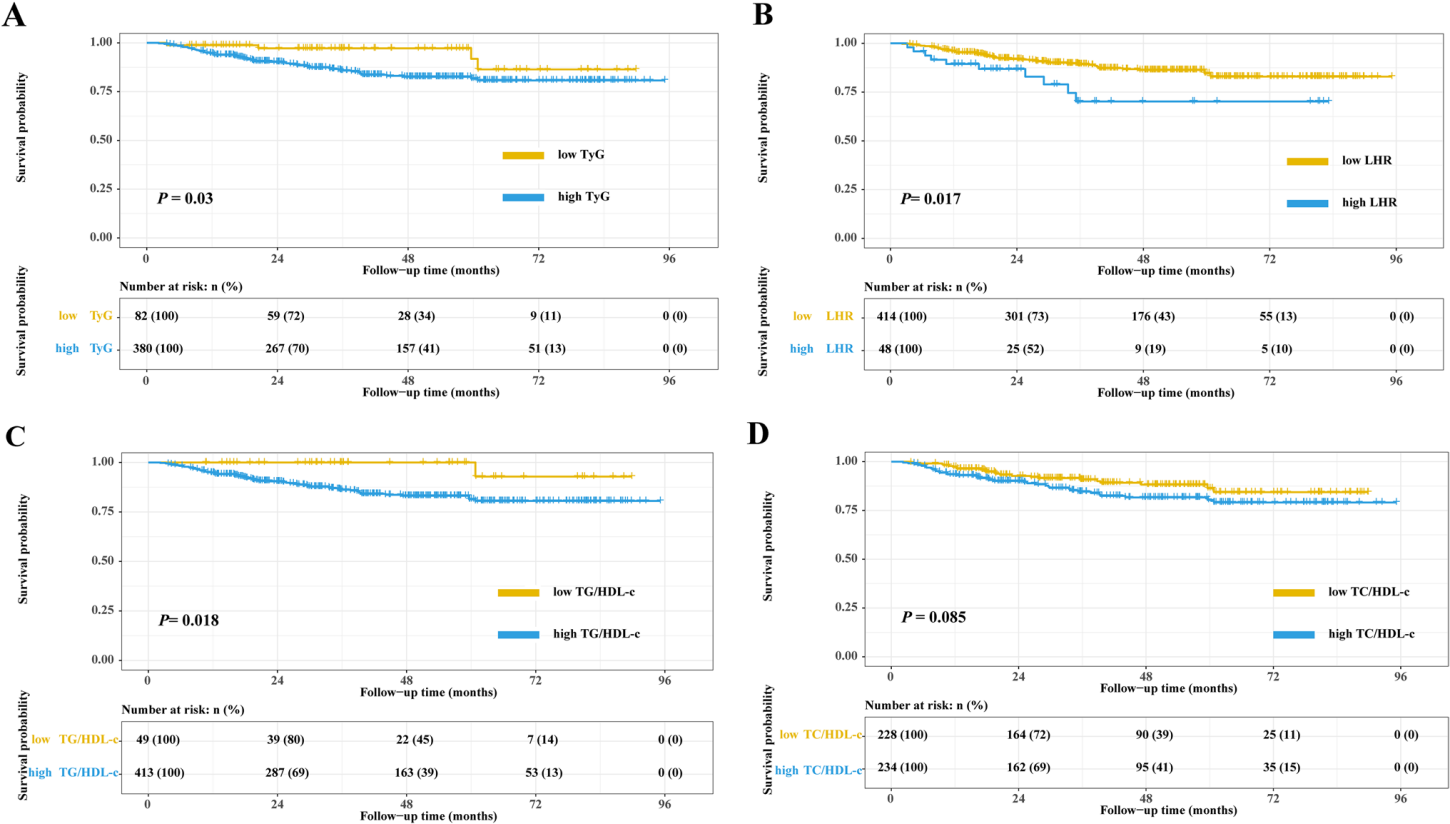
The Kaplan–Meier survival curves of IR makers in women with breast cancer. (**A**) TyG; (**B**) LHR; (**C**) TG/HDL-c; (**D**) TC/HDL-c. *Notes IR* insulin resistance, *TyG* triglyceride-glucose index, *LHR* LDL-c/HDL-c ratio, *HDL-c* high-density lipoprotein cholesterol, *LDL-c* low-density lipoprotein cholesterol.

The survival curves of CRP, CAR, and LCR in women with breast cancer showed that patients with high CRP, high CAR, or high LCR had a worse prognosis than patients with low CRP(*P* = 0.0025), low CAR (*P* < 0.001), or low LCR (*P* < 0.001), respectively (Fig. 4A–C). In addition, survival curves showed that compared with patients with low TyG, low LHR, or low TG/HDL-c, patients with high TyG (*P* = 0.03), high LHR (*P* = 0.017), or high TG/HDL-c (*P* = 0.018) had worse prognosis, respectively. However, there was no significant difference in survival between patients with low TC/HDL-c or high TC/HDL-c (*P* = 0.085) (Fig. 5A–D).

Multivariate survival analysis of systemic inflammatory indicators in women with breast cancer indicated that patients with high CRP [model 4: HR (95% CI) = 2.21 (1.24–3.94), *P* = 0.007] had a shorter OS than patients with low CRP, patients with high CAR [model 4: HR (95% CI) = 2.56 (1.46–4.47), *P* = 0.001] had a shorter OS than those with low CAR, and patients with high LCR [model 4: HR (95% CI) = 2.43 (1.47–4.02), *P* = 0.001] had a shorter OS than patients with low LCR (Table 2).

**Table 2:** Univariate and multivariate analysis.

Variables	OS (model 0)		OS (model 1)		OS (model 2)		OS (model 3)		OS (model 4)	
	Crude HR (95% CI)	Crude *P*	Adjusted HR (95% CI)	Adjusted *P*	Adjusted HR (95% CI)	Adjusted *P*	Adjusted HR (95% CI)	Adjusted *P*	Adjusted HR (95% CI)	Adjusted *P*
CRP										
As continues	1.12 (0.91–1.37)	0.288	1.12 (0.91–1.37)	0.290	1.15 (0.92–1.44)	0.231	1.20 (0.96–1.50)	0.115	1.20 (0.96–1.51)	0.109
As binary										
CRP ≤ 10	Ref		Ref		Ref		Ref		Ref	
CRP > 10	3.37 (1.93–5.88)	< 0.001	2.99 (1.73–5.16)	< 0.001	1.8 (1.03–3.14)	0.039	2.21 (1.24–3.94)	0.007	2.21 (1.24–3.94)	0.007
As tertiles										
T1 (< 0.170)	Ref		Ref		Ref		Ref		Ref	
T2 (0.170–0.528)	0.98 (0.52–1.84)	0.946	0.96 (0.51–1.82)	0.906	0.77 (0.41–1.45)	0.416	0.77 (0.40–1.48)	0.441	0.77 (0.40–1.48)	0.439
T3 (> 0.528)	1.84 (1.07–3.19)	0.029	1.81 (1.04–3.15)	0.037	1.18 (0.67–2.06)	0.565	1.25 (0.70–2.23)	0.457	1.26 (0.71–2.26)	0.433
*P* for trend		0.024		0.029		0.478		0.377		0.358
CAR	Ref		Ref		Ref		Ref		Ref	
As continues	1.13 (0.91–1.42)	0.275	1.13 (0.91–1.42)	0.273	1.14 (0.90–1.45)	0.285	1.18 (0.93–1.50)	0.179	1.18 (0.93–1.51)	0.169
As binary										
CAR ≤ 0.24										
CAR > 0.24	3.2 (1.89–5.42)	< 0.001	3.21 (1.9–5.43)	< 0.001	2.05 (1.2–3.52)	0.009	2.56 (1.46–4.47)	0.001	2.56 (1.46–4.47)	0.001
As tertiles										
T1 (< 0.034)	Ref		Ref		Ref		Ref		Ref	
T2 (0.034–0.087)	1.07 (0.57–2.00)	0.84	1.05 (0.55–1.97)	0.89	0.78 (0.41–1.46)	0.433	0.78 (0.41–1.50)	0.461	0.78 (0.41–1.50)	0.462
T3 (> 0.087)	1.98 (1.13–3.45)	0.016	1.94 (1.11–3.41)	0.021	1.15 (0.65–2.04)	0.635	1.27 (0.70–2.30)	0.432	1.28 (0.71–2.32)	0.415
*P* for trend		0.013		0.016		0.526		0.346		0.332
LCR										
As continues	1.10 (0.52–2.30)	0.808	1.12 (0.54–2.35)	0.762	1.45 (0.66–3.16)	0.357	1.48 (0.69–3.15)	0.315	1.46 (0.69–3.12)	0.324
As binary										
LCR ≤ 2321.9	Ref		Ref		Ref		Ref		Ref	
LCR > 2321.9	3.46 (2.17–5.52)	< 0.001	3.44 (2.15–5.50)	< 0.001	2.03 (1.25–3.32)	0.004	2.44 (1.47–4.03)	0.001	2.43 (1.47–4.02)	0.001
As tertiles										
T1 (> 10,608.11)	Ref		Ref		Ref		Ref		Ref	
T2 (4000–10,608.11)	1.09 (0.57–2.1)	0.794	1.08 (0.56–2.08)	0.819	0.9 (0.47–1.73)	0.745	0.9 (0.46–1.78)	0.765	0.91 (0.46–1.79)	0.782
T3 (< 4000)	2.43 (1.38–4.29)	0.002	2.4 (1.36–4.26)	0.003	1.34 (0.75–2.4)	0.322	1.47 (0.81–2.7)	0.209	1.47 (0.81–2.7)	0.209
*P* for trend		0.001		0.002		0.261		0.159		0.161
TyG										
As continues	1.08 (0.85–1.37)	0.534	1.07 (0.84–1.36)	0.587	1.03 (0.81–1.31)	0.84	0.97 (0.75–1.25)	0.789	0.97 (0.75–1.25)	0.805
As binary										
TyG ≤ 4.72	Ref		Ref		Ref		Ref		Ref	
TyG > 4.72	1.89 (1.06–3.40)	0.032	1.86 (1.03–3.35)	0.04	1.56 (0.85–2.87)	0.149	1.38 (0.71–2.68)	0.343	1.42 (0.73–2.78)	0.302
As tertiles										
T1 (< 4.459)	Ref		Ref		Ref		Ref		Ref	
T2 (4.459–4.584)	0.81 (0.45–1.48)	0.497	0.79 (0.43–1.45)	0.454	1.01 (0.55–1.84)	0.976	1.00 (0.54–1.86)	0.993	1.00 (0.54–1.87)	0.993
T3 (> 4.584)	1.15 (0.67–2.00)	0.607	1.12 (0.64–1.95)	0.683	1.03 (0.59–1.8)	0.907	0.94 (0.51–1.74)	0.835	0.94 (0.51–1.74)	0.850
*P* for trend		0.571		0.636		0.906		0.833		0.848
LHR										
As continues	1.18 (0.94–1.47)	0.159	1.16 (0.92–1.47)	0.196	1.05 (0.83–1.33)	0.708	1.04 (0.82–1.33)	0.735	1.04 (0.82–1.33)	0.740
As binary										
LHR ≤ 3.20	Ref		Ref		Ref		Ref		Ref	
LHR > 3.20	2.18 (1.17–4.06)	0.014	2.16 (1.16–4.02)	0.016	2.02 (1.08–3.79)	0.028	2.42 (1.27–4.63)	0.008	2.40 (1.25–4.61)	0.008
As tertiles										
T1 (< 1.925)	Ref		Ref		Ref		Ref		Ref	
T2 (1.925–2.554)	0.91 (0.51–1.63)	0.752	0.90 (0.50–1.61)	0.719	0.84 (0.47–1.51)	0.552	0.89 (0.49–1.62)	0.696	0.87 (0.47–1.59)	0.649
T3 (> 2.554)	1.13 (0.65–1.98)	0.659	1.10 (0.62–1.93)	0.751	0.93 (0.53–1.64)	0.796	0.86 (0.48–1.54)	0.612	0.86 (0.48–1.54)	0.607
*P* for trend		0.646		0.738		0.813		0.617		0.616
TG/HDL										
As continues	0.97 (0.77–1.21)	0.771	0.95 (0.76–1.20)	0.693	0.99 (0.77–1.28)	0.952	0.91 (0.71–1.17)	0.478	0.92 (0.71–1.18)	0.495
As binary										
TG/HDL-c ≤ 0.60	Ref		Ref		Ref		Ref		Ref	
TG/HDL-c > 0.60	4.31 (1.36–13.69)	0.013	4.25 (1.33–13.56)	0.015	3.84 (1.20–12.32)	0.24	3.50 (1.08–11.32)	0.370	3.51 (1.08–11.36)	0.036
As tertiles										
T1 (< 0.912)	Ref		Ref		Ref		Ref		Ref	
T2 (0.912–1.621)	1.64 (0.91–2.96)	0.098	1.61 (0.88–2.94)	0.123	1.24 (0.68–2.29)	0.484	1.31 (0.71–2.44)	0.388	1.33 (0.71–2.47)	0.372
T3 (> 1.621)	1.33 (0.73–2.44)	0.356	1.30 (0.69–2.43)	0.415	1.13 (0.60–2.10)	0.705	1.10 (0.58–2.09)	0.769	1.12 (0.59–2.14)	0.731
*P* for trend		0.393		0.484		0.757		0.819		0.781
TC/HDL										
As Continues	1.14 (0.90–1.46)	0.279	1.13 (0.88–1.45)	0.338	1.10 (0.83–1.46)	0.508	1.04 (0.79–1.36)	0.782	1.04 (0.79–1.36)	0.785
As binary										
TC/HDL-c ≤ 3.81	Ref		Ref		Ref		Ref		Ref	
TC/HDL-c > 3.81	1.75 (1.09–2.82)	0.02	1.73 (1.07–2.81)	0.025	1.38 (0.85–2.23)	0.195	1.38 (0.84–2.26)	0.204	1.40 (0.85–2.30)	0.185
As tertiles										
T1 (< 3.335)	Ref		Ref		Ref		Ref		Ref	
T2 (3.335–4.158)	1.13 (0.63–2.03)	0.682	1.11 (0.61–2.00)	0.735	1.03 (0.57–1.86)	0.925	1.02 (0.56–1.86)	0.947	1.02 (0.56–1.87)	0.942
T3 (> 4.158)	1.27 (0.72–2.24)	0.414	1.22 (0.68–2.20)	0.498	1.08 (0.61–1.93)	0.787	1.04 (0.57–1.88)	0.909	1.05 (0.58–1.90)	0.885
*P* for trend		0.413		0.497		0.785		0.909		0.885

*CRP* C-reactive protein, *CAR* CRP/Albumin ratio, *LCR* lymphocyte/CRP ratio, *TyG* fasting triglyceride glucose index, *TG* triglyceride, *TC* total cholesterol, *LHR* LDL-c/HDL-c ratio, *HDL-c* high-density lipoprotein cholesterol, *LDL-c* low-density lipoprotein cholesterol, *HR* hazards ratio, *CI* confidence interval, *BMI* body mass index, *KPS* karnofsky performance status, *TSF* triceps skinfold thickness.

^a^Model 0: Unadjusted.

^b^Model 1: Adjusted for BMI.

^c^Model 2: Adjusted for age, BMI and tumor stage.

^d^Model 3: Adjusted for age, tumor stage, BMI, KPS, surgery, chemotherapy, radiotherapy, immunotherapy, family history of cancer, tumor metastasis, diabetes, hypertension, and coronary heart disease.

^e^Model 4: Adjusted for age, tumor stage, BMI, KPS, surgery, chemotherapy, radiotherapy, immunotherapy, family history of cancer, tumor metastasis, diabetes, hypertension, coronary heart disease, and TSF.

Multivariate survival analysis of the IR index in women with breast cancer indicated that patients with high LHR [model 4: HR (95% CI) = 2.40 (1.25–4.61), *P* = 0.008] had a shorter OS than those with low LHR and patients with high TG/ HDL-c [model 4: HR (95% CI) = 3.51 (1.08–11.36), *P* = 0.036] had a shorter OS than those with low TG/HDL-c. However, TyG [model 4: HR (95% CI) = 1.42 (0.73–2.78), *P* = 0.302] and TC/HDL-c [model 4: HR (95% CI) = 1.40 (0.85–2.30), *P* = 0.185] were not significant survival predictors in women with breast cancer (Table 2).

### Survival analysis stratified by different BMI groups

We analyzed the prognostic value of systemic inflammatory markers and IR markers in different BMI subgroups. In the BMI < 24 kg/m^2^ subgroup, we observed that all markers did not show significant prognostic value (All *P* > 0.05). In the BMI ≥ 24 kg/m^2^ subgroup, patients with high CRP [Adjusted HR (95% CI) = 2.39 (1.00–5.71), *P* = 0.049], high CAR [Adjusted HR (95% CI) = 2.85 (1.23–6.60), *P* = 0.014], high LCR [Adjusted HR (95% CI) = 4.32 (2.06–9.06), *P* < 0.001], high TyG [Adjusted HR (95% CI) = 2.87 (1.20–6.85), *P* = 0.017], or high LHR [Adjusted HR (95% CI) = 2.91 (1.20–7.06), *P* = 0.018] predicted worse prognoses, while TG/HDL-c and TC/HDL-c did not show significant prognostic value (All *P* > 0.05) (Table 3).

**Table 3:** Survival analysis stratified by different BMI groups.

Variables	BMI < 24 (kg/m^2^)*	*P* value	BMI > 24 (kg/m^2^)*	*P* value
CRP				
CRP ≤ 10	1		1	
CRP > 10	1.81 (0.78–4.23)	0.170	2.39 (1.00–5.71)	0.049
CAR				
CAR ≤ 0.24				
CAR > 0.24	2.06 (0.90–4.73)	0.089	2.85 (1.23–6.60)	0.014
LCR				
LCR ≤ 2321.9				
LCR > 2321.9	1.44 (0.67–3.12)	0.350	4.32 (2.06–9.06)	< 0.001
TyG				
TyG ≤ 4.72	1		1	
TyG > 4.72	0.55 (0.16–1.94)	0.350	2.87 (1.20–6.85)	0.017
LHR				
LHR ≤ 3.20	1		1	
LHR > 3.20	2.06 (0.70–6.10)	0.192	2.91 (1.20–7.06)	0.018
TGH				
TG/HDL-c ≤ 0.60	1		1	
TG/HDL-c > 0.60	3.78 (0.87–16.44)	0.077	2.61 (0.34–19.92)	0.355
TCH				
TC/HDL-c ≤ 3.81	1		1	
TC/HDL-c > 3.81	0.62 (0.26–1.48)	0.285	1.58 (0.77–3.25)	0.211

*CRP* C-reactive protein, *LHR* LDL-c/HDL-c ratio, *HDL-c* high-density lipoprotein cholesterol, *LDL-c* low-density lipoprotein cholesterol, *HR* hazards ratio, *CI* confidence interval, *BMI* body mass index, *KPS* karnofsky performance status, *TSF* triceps skinfold thickness.

*Adjusted for age, tumor stage, KPS, surgery, chemotherapy, radiotherapy, immunotherapy, family history of cancer, tumor metastasis, diabetes, hypertension, coronary heart disease, and TSF.

### Combined analysis of prognostic systemic inflammatory indicators and IR index

We performed a combined survival analysis with the prognostic systemic inflammatory index and IR index. In all patients, CAR combined with LHR or LCR combined with LHR predicted a longer OS in women with breast cancer. The prognosis of patients in the low CAR and high LHR or high CAR and low LHR group [Adjusted HR (95% CI) = 2.21 (1.27–3.87), *P* = 0.005] and the high CAR and high LHR group [Adjusted HR (95% CI) = 3.91 (1.56–9.81), *P* = 0.004] was worse than in patients in the low CAR and low LHR groups. The prognosis of patients in the high LCR and high LHR or low LCR and low LHR group [Adjusted HR (95% CI) = 2.30 (1.36–3.87), *P* = 0.002] and the low LCR and high LHR group [Adjusted HR (95% CI) = 3.89 (1.65–9.21), *P* = 0.002] was worse than in patients in the high LCR and low LHR groups. However, no prognostic information was generated by the other combinations. In addition, when we performed a combined survival analysis in different BMI subgroups, we only observed a significant survival difference in the combined analysis of LCR and LHR in the high BMI subgroup. The prognosis of patients in the high LCR and high LHR or low LCR and low LHR group [Adjusted HR (95% CI) = 3.61 (1.69–7.69), *P* = 0.001] and the low LCR and high LHR group [Adjusted HR (95% CI) = 7.79 (2.42–25.11), *P* = 0.001] was worse than that of the patients in the low LCR and low LHR groups (Table 4).

**Table 4:** Combined survival analysis of prognostic systemic and IR indicators.

Variables	All patients#		BMI < 24 (kg/m^2^)*		BMI ≥ 24 (kg/m^2^)*	
	Adjusted HR (95% CI)	*P* value	Adjusted HR (95% CI)	*P* value	Adjusted HR (95% CI)	*P* value
CRP and LHR						
Low CRP and low LHR	1		1		1	
Low CRP and high LHR or high CRP and low LHR	2.48 (1.44–4.27)	0.001	2.23 (0.95–5.27)	0.066	2.42 (1.15–5.09)	0.020
High CRP and high LHR	2.56 (0.88–7.51)	0.086	1.93 (0.39–9.54)	0.419	4.75 (0.97–23.3)	0.055
*P* for trend		0.003		0.139		0.005
CAR and LHR						
Low CAR and low LHR	1		1		1	
Low CAR and high LHR or high CAR and low LHR	2.21 (1.27–3.87)	0.005	2.05 (0.85–4.94)	0.111	2.13 (0.99–4.58)	0.054
High CAR and high LHR	3.91 (1.56–9.81)	0.004	2.68 (0.66–10.88)	0.168	8.80 (2.19–35.31)	0.002
*P* for trend		< 0.001		0.056		0.001
LCR and LHR						
High LCR and low LHR	1		1		1	
Low LCR and low LHR or high LCR and high LHR	2.30 (1.36–3.87)	0.002	1.44 (0.64–3.25)	0.382	3.61 (1.69–7.69)	0.001
Low LCR and high LHR	3.89 (1.65–9.21)	0.002	2.32 (0.59–9.14)	0.231	7.79 (2.42–25.11)	0.001
*P* for trend		< 0.001		0.175		< 0.001
CRP and TG/HDL-c						
Low CRP and Low TG/HDL-c	1		1		1	
Low CRP and high TG/HDL-c or high CRP and low TG/HDL-c	2.64 (0.81–8.62)	0.109	2.55 (0.57–11.52)	0.223	2.30 (0.3–17.71)	0.424
High CRP and high TG/HDL-c	5.91 (1.69–20.61)	0.005	5.33 (1.07–26.69)	0.042	5.44 (0.62–47.42)	0.126
*P* for trend		0.001		0.021		0.032
CAR and TG/HDL-c						
Low CAR and low TG/HDL-c	1		1		1	
Low CAR and high TG/HDL-c or high CAR and low TG/HDL-c	2.52 (0.77–8.25)	0.127	2.46 (0.54–11.13)	0.242	2.24 (0.29–17.32)	0.438
High CAR and high TG/HDL-c	6.50 (1.88–22.44)	0.003	5.84 (1.18–28.92)	0.031	6.32 (0.73–54.51)	0.094
*P* for trend		< 0.001		0.011		0.011
LCR and TG/HDL-c						
High LCR and low TG/HDL-c	1		1		1	
Low LCR and low TG/HDL-c or high LCR and high TG/HDL-c	2.18 (0.66–7.17)	0.200	2.34 (0.51–10.7)	0.273	1.56 (0.2–12.24)	0.673
Low LCR and high TG/HDL-c	5.55 (1.64–18.72)	0.006	3.79 (0.79–18.11)	0.095	6.69 (0.84–53.17)	0.072
*P* for trend		< 0.001		0.062		< 0.001

*CRP* C-reactive protein, *LHR* LDL-c/HDL-c ratio, *HDL-c* high-density lipoprotein cholesterol, *LDL-c* low-density lipoprotein cholesterol, *HR* hazards ratio, *CI* confidence interval, *BMI* body mass index, *KPS* karnofsky performance status, *TSF* triceps skinfold thickness.

*Adjusted for age, tumor stage, KPS, surgery, chemotherapy, radiotherapy, immunotherapy, family history of cancer, tumor metastasis, diabetes, hypertension, coronary heart disease, and TSF.

## Discussion

In this study, we found that the levels of inflammation (CRP, CAR, and LCR) and IR (TyG, LHR, TG/HDL-c, and TC/HDL-c) in breast cancer patients with BMI ≥ 24 kg/m^2^ were significantly higher than those in patients with BMI < 24 kg/m^2^. In other words, inflammation and IR levels in overweight or obese patients are high. The expression of adipocytokines in human adipose tissue and their corresponding circulating concentrations are influenced by human fat mass. In obese patients, there was also a positive correlation between adipocyte TNF-α expression and plasma TNF-α concentration with BMI^40^. Plasma IL-6 and CRP concentrations were also positively correlated with BMI^23,41^. Obesity is a common cause of chronic inflammation, and white adipose tissue (WAT) in obese patients is infiltrated by immune cells, including macrophages and lymphocytes, at the systemic and tissue level. WAT inflammation is associated with increased circulating levels of CRP and IL-6^16^. Obesity is a well-established risk factor for IR and type II diabetes. With the rising prevalence of obesity, an increasing number of patients at cancer diagnosis are overweight or obese and have impaired glycemic control. Obesity and excess adipose tissue lead to increased production of free fatty acids, leptin, and cytokines, and these metabolic abnormalities are associated with decreased physical activity and increased triglycerides, leading to hyperinsulinemia and IR^42^.

We also examined the relationship between systemic inflammation and IR and breast cancer survival. Studies have shown an increased risk of breast cancer in obese postmenopausal women, and it has been hypothesized that circulating estrogen levels may be elevated in obese postmenopausal women^43^. Therefore, considering these potential interference factors, we separated patients into different age groups and adjusted the survival analyses to reduce the interference caused by estrogen levels in different age groups. We found that elevated systemic inflammatory markers (CRP, CAR, and LCR) were all significantly associated with reduced OS in breast cancer patients. Similarly, we observed a significant association between increased IR markers (LHR and TG/ HDL-c) and decreased OS in breast cancer patients.

Pierce et al. analyzed the prognostic value of inflammatory markers in women with stage 0 to IIIA breast cancer in a multicenter, prospective, cohort study and found that CRP was associated with poor prognosis in women with breast cancer compared with the highest and lowest tertiles [HR 2.27; 95% CI 1.27–4.08]^26^. Gunter et al. and Albuquerque et al. also found that CRP levels were positively associated with breast cancer risk^27,28^. Zhou et al. used propensity score matching to estimate the prognostic role of CAR in non-metastatic breast cancer patients and found that elevated CAR levels were associated with increased age, postmenopausal status, and a higher risk of recurrence or death in breast cancer patients. Elevated CAR was an independent risk factor for long-term prognosis, predicting decreased disease-free survival [HR 2.225; *P* = 0.024] and OS [HR 9.189; *P* = 0.003] of breast cancer patients^29^. Chen et al. found that preoperative CAR could be an important independent prognostic marker for HER2-negative, luminal breast cancer, and elevated CAR was associated with poorer disease-free survival and cancer-specific survival^30^. In this study, for the first time, we found that LCR could be used as an independent prognostic marker in breast cancer patients. Previous studies reported that LCR is associated with poor prognosis in other tumors, such as colorectal cancer^33^, gastric cancer^32^, and hepatocellular carcinoma^44^. As for IR prognostic indicators, previous studies have shown that LHR is associated with poor prognosis in colorectal cancer^45,46^ and gastric cancer^47^. Dai et al. analyzed the relationship between TG/ HDL-c and prognosis in triple-negative breast cancer patients and found that patients with high TG/ HDL-c was associated with poor OS [HR: 1.935; 95% CI 1.032–3.629]^48^. Similar results showed that TG/ HDL-c was associated with poor prognosis in other cancers, including in endometrial cancer^49^ and gastric cancer^50^.

We observed markers of inflammation and IR in different BMI subgroups and found that LCR could predict survival in different BMI subgroups. And CRP, CAR, TyG, LHR predict the prognosis of patients within the high BMI subgroup. The results of the combined survival analyses showed that the inflammatory insulin combination of LCR&LHR and CAR&LHR could differentiate the prognosis of breast cancer patients. Especially, LCR&LHR could also significantly differentiate the prognosis of patients in the high BMI subgroup. Furthermore, the observation that breast WAT inflammation predicts a poorer clinical course in breast cancer patients is consistent with earlier reports showing that TNF-α , IL-1beta, IL-6 and CRP promote tumor growth in a mouse model of obesity and elevated levels of IL-6 and CRP were associated with the development and progression with female breast cancer^16^. Inflammation and IR are closely related. The IR state that develops with increased obesity is associated with activation of inflammatory responses in different organ sites, including adipose tissue, liver, and skeletal muscle, which increases secretion and systemic levels of proinflammatory cytokines^51^. Some adipocytokines help regulate insulin action and are associated with IR syndromes^52^. Leptin interferes with insulin signaling, and in type II diabetes, plasma leptin levels correlate with the degree of IR, a relationship independent of BMI and body fat mass^53,54^. Thus, the IR syndrome is associated with hyperleptinemia and hyperinsulinemia^55^, which allows endocrine hyperactivity of these proteins at target sites, including mammary epithelial tissue and vascular endothelial cells. Adipose tissue TNF-α expression was also positively correlated with plasma insulin concentrations^56^, and increased adipocyte secretion of TNF-α was associated with decreased insulin sensitivity in obese individuals^41^. In abdominal obesity, high circulating TNF-α levels are associated with hyperinsulinemia and IR^57^. IR is also associated with human adipose tissue-derived IL-6^41^. Adipose tissue has biological activities that regulate appetite, inflammation, insulin sensitivity, fat metabolism, and energy balance^58^. Excessive adipose tissue will lead to the production of inflammatory cytokines and the upregulation of nuclear factor-κB, leading to increased nitric oxide and reactive oxygen species, resulting in IR, excess glucose, and increased free fatty acid, thereby further spreading inflammation^59^.

Our study has several strengths. First, this is a prospective, cohort study of women with breast cancer based on a multi-medical center trial to analyze the prognosis of different systemic inflammation and IR markers. Second, our study analyzed the inflammation and IR levels in different BMI subgroups and examined high-inflammation and high-IR status in overweight or obese female breast cancer patients to identify the best markers of inflammation and IR. Our study also has some limitations. First, we only collected a fasting blood sample and thus, cross-sectional data. Longitudinal data is needed for a patient's observation of inflammation and IR. Second, although we consider the effect of hormonal levels in patients and make prognostic adjustments for different ages, we still need to collect relevant data. Third, different pathological types of breast cancer may cause heterogeneity, and the results of more pathological types need to be included. Fourth, our IR-related metric is only a surrogate metric, and we cannot deny its simplicity and feasibility, but the assessment of patients' IR status still needs to be done.

## Conclusion

In conclusion, our data showed that higher CRP, CAR, LCR, LHR, and TG/ HDL-c were associated with increased risk in women with breast cancer. Elevated BMI showed the higher inflammation and IR levels in women with breast cancer. The combination of CAR and LHR or LCR and LHR could significantly predict the prognosis of women with breast cancer, while the combination of LCR and LHR can significantly predict prognosis in those patients with overweight or obese patients.

## Materials and methods

### Study population

The data collected in this study from women with breast cancer were obtained from a prospective, multi-medical center-based cancer population study in China between 2013 and 2021. The hospitals included Fujian Cancer Hospital, Bethune First Hospital of Jilin University, Zhejiang Cancer Hospital, First Affiliated Hospital of Sun Yat-Sen University, Chongqing Daping Hospital, and Chongqing Third People's Hospital. The inclusion criteria for this study were: 1. Female patients aged not less than 18 years; 2. Pathologically diagnosed with breast cancer; and 3. Clearly conscious and able to communicate autonomously. There were no strict exclusion criteria. The current study complied with the Declaration of Helsinki, was approved by the Human Research Committees at the various medical centers, and all participants provided informed consent.

### Anthropometric and laboratory measurements

At the start of the study, participants' demographic information, medical and family history, and quality of life assessment were collected through questionnaires administered by trained investigators. All research centers, which participated in your study, had the same standards of biomarkers laboratory testing. Baseline clinical characteristics collected from patients included age, body mass index (BMI), comorbidities (diabetes, yes/no; hypertension, yes/no; and coronary heart disease, yes/no), tumor-related information (family history of cancer, tumor stage, surgery, yes/no; radiation therapy, yes/no; chemotherapy, yes/no; immunotherapy, yes/no; and tumor metastasis, yes/no), Karnofsky performance status (KPS), triceps skinfold thickness (TSF), and laboratory test indicators [C-reactive protein (CRP), fasting blood glucose (FBG), triglyceride (TG), total cholesterol (TC), low-density lipoprotein cholesterol (LDL-c), and high-density lipoprotein cholesterol (HDL-c)].

The patient's body measurements were obtained by clinicians or nurses, height and weight were measured while the patients were wearing light hospital gowns and socks, and TSF was obtained by taking the average of three measurements with a skinfold caliper. BMI was defined as the ratio of weight (kg) to height squared (m^2^). Blood samples from patients were collected for analysis in the laboratory within 48 h prior to admission after patients had fasted for at least 8 h prior to sample collection. The index of CAR and LCR were calculated by: CRP/albumin and Lymphocyte/CRP, respectively. The TyG index was defined as Ln [TC (mg/dl) * FBG (mg/dl)]/2. The ratios LDL-c/HDL-c (LHR), TG/HDL-c, and TC/HDL-c were defined as: LDL-c/HDL-c, TG/HDL-c, and TC/HDL-c, respectively.

### Outcomes

Overall survival (OS), representing the study endpoint, was calculated from the date of diagnosis of cancer until death or last follow-up. Follow-up of patients was completed by follow-up staff.

### Statistical analyses

Data are shown as percentages, mean ± standard deviation, or median ± interquartile interval. Baseline characteristics of obese and nonobese populations were compared using the chi-square test and Fisher's exact test for categorical variables and a t-test for continuous normal distribution variables (Wilcoxon test for non-parametric variables). Cutoff values were generated by largest selected rank statistical analysis method for continuous data (see Supplementary Fig. S1 online).

The prognostic AUC curves were performed to selcet the optimal inflammation index and IR index. The survival curves were calculated using the Kaplan–Meier method, and the level of significance was assessed using the log-rank test. Associations between prognostic factors and OS were examined using multivariable Cox proportional hazards regression models, and results were reported as hazard ratios (HRs) and 95% confidence intervals (95% CIs). We assessed confounding covariates by adding each covariate sequentially to the base model. Model 0: unadjusted; model 1: adjusted for BMI; model 2: adjusted for age, tumor stage, and BMI; model 3: adjusted for age, tumor stage, BMI, KPS, surgery, chemotherapy, radiotherapy, immunotherapy, family history of cancer, tumor metastasis, diabetes, hypertension, and coronary heart disease.; model 4: adjusted for age, tumor stage, BMI, KPS, surgery, chemotherapy, radiotherapy, immunotherapy, family history of cancer, tumor metastasis, diabetes, hypertension, coronary heart disease, and TSF.

All *P* values were two-sided. *P* values less than 0.05 were considered statistically significant. All statistical analyses were performed using the R software version 4.1.1.

### Ethics approval

This study followed the Helsinki declaration. All participants signed an informed consent form, and this study was approved by the Institutional Review Board of each hospital (Registration number: ChiCTR1800020329).

## Supplementary Information

Supplementary Figure S1.

## Data Availability

The datasets generated during and/or analysed during the current study are available from the corresponding author on reasonable request.

## References

[1] Siegel RL, Miller KD, Fuchs HE, Jemal A (2022). Cancer statistics, 2022. CA Cancer J. Clin..

[2] Chen W (2016). Cancer statistics in China, 2015. CA Cancer J. Clin..

[3] Lee DY (2018). Impact of systemic inflammation on the relationship between insulin resistance and all-cause and cancer-related mortality. Metabolism.

[4] Korniluk A, Koper O, Kemona H, Dymicka-Piekarska V (2017). From inflammation to cancer. Ir. J. Med. Sci..

[5] Argiles JM, Lopez-Soriano FJ, Busquets S (2012). Counteracting inflammation: A promising therapy in cachexia. Crit. Rev. Oncog..

[6] Fearon KC (1999). Pancreatic cancer as a model: Inflammatory mediators, acute-phase response, and cancer cachexia. World J. Surg..

[7] Argiles JM, Lopez-Soriano FJ (2001). Insulin and cancer (Review). Int. J. Oncol..

[8] Tayek JA (1992). A review of cancer cachexia and abnormal glucose metabolism in humans with cancer. J. Am. Coll. Nutr..

[9] Glicksman AS, Rawson RW (1956). Diabetes and altered carbohydrate metabolism in patients with cancer. Cancer.

[10] Dev R, Bruera E, Dalal S (2018). Insulin resistance and body composition in cancer patients. Ann. Oncol..

[11] Fonseca G, Farkas J, Dora E, von Haehling S, Lainscak M (2020). Cancer cachexia and related metabolic dysfunction. Int. J. Mol. Sci..

[12] Formiguera X, Canton A (2004). Obesity: Epidemiology and clinical aspects. Best Pract. Res. Clin. Gastroenterol..

[13] Finucane MM (2011). National, regional, and global trends in body-mass index since 1980: Systematic analysis of health examination surveys and epidemiological studies with 960 country-years and 9.1 million participants. Lancet..

[14] Duggan C (2011). Associations of insulin resistance and adiponectin with mortality in women with breast cancer. J. Clin. Oncol..

[15] Majed B, Moreau T, Asselain B, Curie Institute Breast Cancer, G (2009). Overweight, obesity and breast cancer prognosis: Optimal body size indicator cut-points. Breast Cancer Res. Treat..

[16] Iyengar NM, Gucalp A, Dannenberg AJ, Hudis CA (2016). Obesity and cancer mechanisms: Tumor microenvironment and inflammation. J. Clin. Oncol..

[17] van Kruijsdijk RC, van der Wall E, Visseren FL (2009). Obesity and cancer: The role of dysfunctional adipose tissue. Cancer Epidemiol. Biomark. Prev..

[18] Kershaw EE, Flier JS (2004). Adipose tissue as an endocrine organ. J. Clin. Endocrinol. Metab..

[19] Sartipy P, Loskutoff DJ (2003). Monocyte chemoattractant protein 1 in obesity and insulin resistance. Proc. Natl. Acad. Sci. USA.

[20] Xu H (2003). Chronic inflammation in fat plays a crucial role in the development of obesity-related insulin resistance. J. Clin. Invest..

[21] Cancello R (2005). Reduction of macrophage infiltration and chemoattractant gene expression changes in white adipose tissue of morbidly obese subjects after surgery-induced weight loss. Diabetes.

[22] Wang Z, Nakayama T (2010). Inflammation, a link between obesity and cardiovascular disease. Mediat. Inflamm..

[23] Amin MN (2019). How the association between obesity and inflammation may lead to insulin resistance and cancer. Diabetes Metab. Syndr..

[24] Guo Q (2015). IGF-I CA19 repeat polymorphisms and cancer risk: A meta-analysis. Int. J. Clin. Exp. Med..

[25] Leung KC, Doyle N, Ballesteros M, Waters MJ, Ho KK (2000). Insulin regulation of human hepatic growth hormone receptors: Divergent effects on biosynthesis and surface translocation. J. Clin. Endocrinol. Metab..

[26] Pierce BL (2009). Elevated biomarkers of inflammation are associated with reduced survival among breast cancer patients. J. Clin. Oncol..

[27] Albuquerque KV (1995). Pre-treatment serum levels of tumour markers in metastatic breast cancer: A prospective assessment of their role in predicting response to therapy and survival. Eur. J. Surg. Oncol..

[28] Gunter MJ (2015). Circulating adipokines and inflammatory markers and postmenopausal breast cancer risk. J. Natl. Cancer Inst..

[29] Zhou L (2019). A retrospective propensity score matched study of the preoperative C-reactive protein to albumin ratio and prognosis in patients with resectable non-metastatic breast cancer. Med. Sci. Monit..

[30] Chen F (2022). Prognostic significance of neutrophil-to-lymphocyte ratio and C-reactive protein/albumin ratio in luminal breast cancers with HER2-negativity. Front. Oncol..

[31] Zhang X (2022). The nutrition-inflammation marker enhances prognostic value to ECOG performance status in overweight or obese patients with cancer. JPEN J. Parenter Enter. Nutr..

[32] Okugawa Y (2020). Lymphocyte-to-C-reactive protein ratio and score are clinically feasible nutrition-inflammation markers of outcome in patients with gastric cancer. Clin. Nutr..

[33] Okugawa Y (2020). Lymphocyte-C-reactive protein ratio as promising new marker for predicting surgical and oncological outcomes in colorectal cancer. Ann. Surg..

[34] Goodwin PJ (2002). Fasting insulin and outcome in early-stage breast cancer: Results of a prospective cohort study. J. Clin. Oncol..

[35] Pasanisi P (2006). Metabolic syndrome as a prognostic factor for breast cancer recurrences. Int. J. Cancer..

[36] DeFronzo RA, Tobin JD, Andres R (1979). Glucose clamp technique: A method for quantifying insulin secretion and resistance. Am. J. Physiol..

[37] Fritz J (2020). The triglyceride-glucose index as a measure of insulin resistance and risk of obesity-related cancers. Int. J. Epidemiol..

[38] Kheirollahi A (2020). Evaluation of lipid ratios and triglyceride-glucose index as risk markers of insulin resistance in Iranian polycystic ovary syndrome women. Lipids Health Dis..

[39] Zhou M (2016). The triglyceride to high-density lipoprotein cholesterol (TG/HDL-C) ratio as a predictor of insulin resistance but not of beta cell function in a Chinese population with different glucose tolerance status. Lipids Health Dis..

[40] Rose DP, Komninou D, Stephenson GD (2004). Obesity, adipocytokines, and insulin resistance in breast cancer. Obes. Rev..

[41] Kern PA, Ranganathan S, Li C, Wood L, Ranganathan G (2001). Adipose tissue tumor necrosis factor and interleukin-6 expression in human obesity and insulin resistance. Am. J. Physiol. Endocrinol. Metab..

[42] Gallagher EJ, LeRoith D (2010). Insulin, insulin resistance, obesity, and cancer. Curr. Diabetes Rep..

[43] Lorincz AM, Sukumar S (2006). Molecular links between obesity and breast cancer. Endocr. Relat. Cancer..

[44] Ni HH (2022). Combining pre- and postoperative lymphocyte-C-Reactive protein ratios can better predict hepatocellular carcinoma prognosis after partial hepatectomy. J. Inflamm. Res..

[45] Liu YL, Qian HX, Qin L, Zhou XJ, Zhang B (2011). Serum LDL-C and LDL-C/HDL-C ratio are positively correlated to lymph node stages in males with colorectal cancer. Hepatogastroenterology..

[46] Notarnicola M (2005). Serum lipid profile in colorectal cancer patients with and without synchronous distant metastases. Oncology.

[47] Ma MZ, Yuan SQ, Chen YM, Zhou ZW (2018). Preoperative apolipoprotein B/apolipoprotein A1 ratio: A novel prognostic factor for gastric cancer. Onco Targets Ther..

[48] Dai D (2016). Pretreatment TG/HDL-C ratio is superior to triacylglycerol level as an independent prognostic factor for the survival of triple negative breast cancer patients. J. Cancer..

[49] Luo YZ (2019). Pretreatment triglycerides-to-high density lipoprotein cholesterol ratio in postmenopausal women with endometrial cancer. Kaohsiung J. Med. Sci..

[50] Sun H (2019). Triglyceride-to-high density lipoprotein cholesterol ratio predicts clinical outcomes in patients with gastric cancer. J. Cancer..

[51] McNelis JC, Olefsky JM (2014). Macrophages, immunity, and metabolic disease. Immunity.

[52] Matsuzawa Y, Funahashi T, Nakamura T (1999). Molecular mechanism of metabolic syndrome X: Contribution of adipocytokines adipocyte-derived bioactive substances. Ann. N. Y. Acad. Sci..

[53] Fischer S (2002). Insulin-resistant patients with type 2 diabetes mellitus have higher serum leptin levels independently of body fat mass. Acta Diabetol..

[54] Wauters M (2003). Leptin levels in type 2 diabetes: Associations with measures of insulin resistance and insulin secretion. Horm. Metab. Res..

[55] Leyva F (1998). Hyperleptinemia as a component of a metabolic syndrome of cardiovascular risk. Arterioscler. Thromb. Vasc. Biol..

[56] Hotamisligil GS, Arner P, Caro JF, Atkinson RL, Spiegelman BM (1995). Increased adipose tissue expression of tumor necrosis factor-alpha in human obesity and insulin resistance. J. Clin. Invest..

[57] Aldhahi W, Hamdy O (2003). Adipokines, inflammation, and the endothelium in diabetes. Curr. Diabates Rep..

[58] Ibrahim MM (2010). Subcutaneous and visceral adipose tissue: Structural and functional differences. Obes. Rev..

[59] Sonnenberg GE, Krakower GR, Kissebah AH (2004). A novel pathway to the manifestations of metabolic syndrome. Obes. Res..

