# Supplementary material for: Comprehensive prognostic effects of systemic inflammation and Insulin resistance in women with breast cancer with different BMI: a prospective multicenter cohort
Source: Sci Rep. 2023 Mar 15;13:4303. doi: 10.1038/s41598-023-31450-w (PMC10017691; doi:10.1038/s41598-023-31450-w)
Supplement: Supplementary file 1 — Supplementary Figure S1. [file 41598_2023_31450_MOESM1_ESM.pdf]

Supplementary Information

Fig. S1

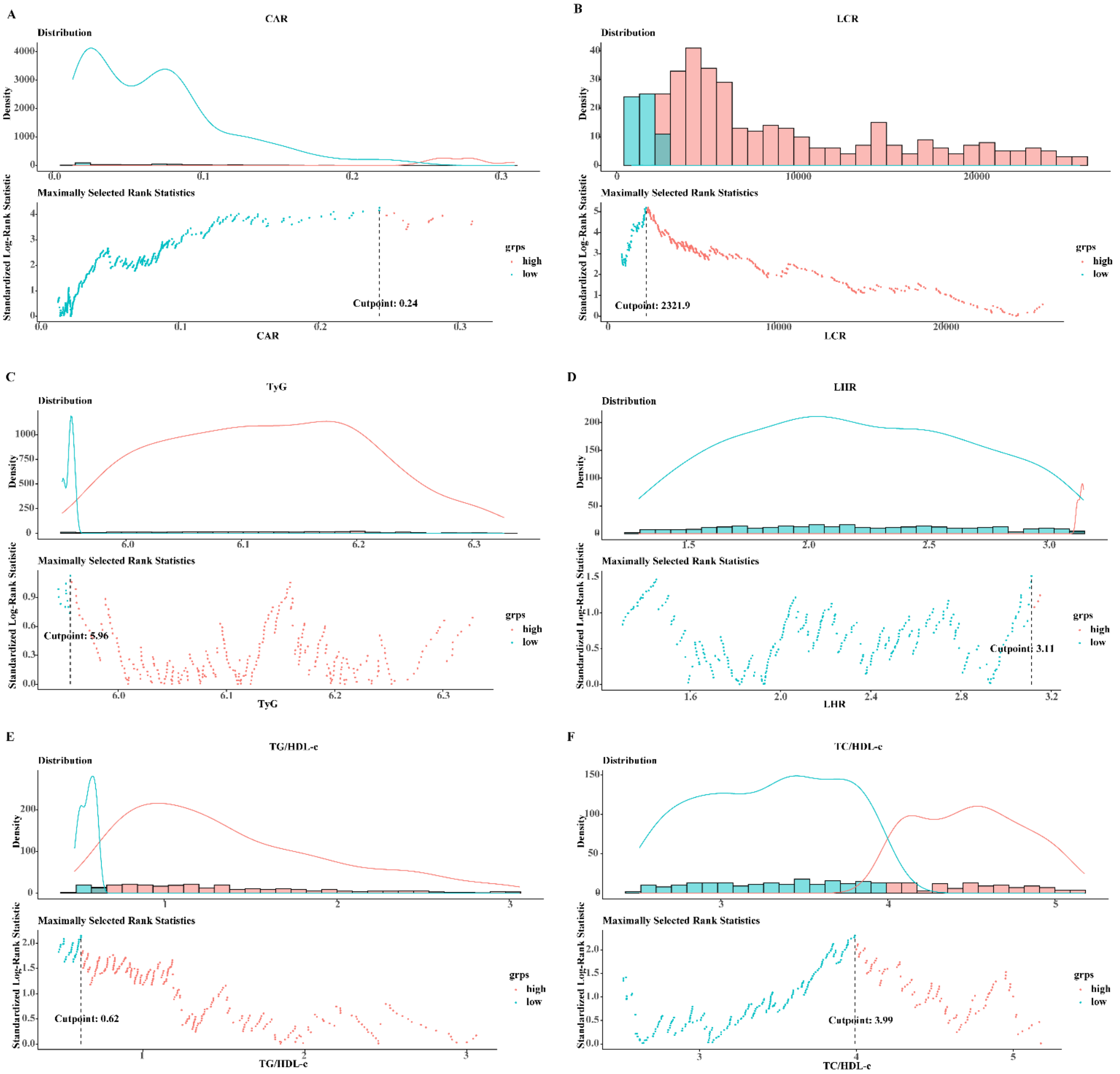

Fig. S1 Optimal cut-off value of LHR.

(A) CAR; (B) LCR; (C) TyG; (D) LHR; (E) TG/HDL-c; (F) TC/HDL-c.

Notes: LCR: lymphocyte to C-reactive protein ratio; CAR: C-reactive protein to albumin ratio; TyG: triglyceride-glucose index; LHR: LDL-c/HDL-c ratio; HDL-c: high-density lipoprotein cholesterol; LDL-c: low-density lipoprotein cholesterol.
